# Supplementary material for: The nuclear envelope protein Net39 is essential for muscle nuclear integrity and chromatin organization
Source: Nat Commun. 2021 Jan 29;12:690. doi: 10.1038/s41467-021-20987-x (PMC7846557; doi:10.1038/s41467-021-20987-x)
Supplement: Supplementary file 1 — Supplementary Information [file 41467_2021_20987_MOESM1_ESM.pdf]

Supplementary Figures for

The nuclear envelope protein Net39 is essential for muscle nuclear integrity and chromatin organization

Andres Ramirez-Martinez[1,2,3†], Yichi Zhang[1,2,3†], Kenian Chen[4], Jiwoong Kim[4], Bercin K. Cenik[1,2,3,7], John R. McAnally[1,2,3], Chunyu Cai[5], John M. Shelton[6], Jian Huang[6], Ana Brennan[1,2,3], Bret M. Evers[5], Pradeep P. A. Mammen[2,3,6], Lin Xu[4], Rhonda Bassel-Duby[1,2,3], Ning Liu[1,2,3*], and Eric N. Olson[1,2,3*]

[1]Department of Molecular Biology, University of Texas Southwestern Medical Center, Dallas, Texas, USA.

[2]Hamon Center for Regenerative Science and Medicine, University of Texas Southwestern Medical Center, Dallas, Texas, USA.

[3]Senator Paul D. Wellstone Muscular Dystrophy Cooperative Research Center, University of Texas Southwestern Medical Center, Dallas, Texas, USA.

[4]Department of Population and Data Sciences; Quantitative Biomedical Research Center University of Texas Southwestern Medical Center, Dallas, Texas, USA.

[5]Department of Pathology, University of Texas Southwestern Medical Center, Dallas, Texas, USA.

[6]Department of Internal Medicine, University of Texas Southwestern Medical Center, Dallas, Texas, USA.

[7]Current address: Simpson Querrey Center for Epigenetics, Department of Biochemistry and Molecular Genetics, Northwestern University Feinberg School of Medicine, 320 East Superior Street, Chicago, IL 60611, USA.

[†] These authors contributed equally.

[*] These authors jointly supervised this work.

Email: Ning.Liu@utsouthwestern.edu; Eric.Olson@utsouthwestern.edu

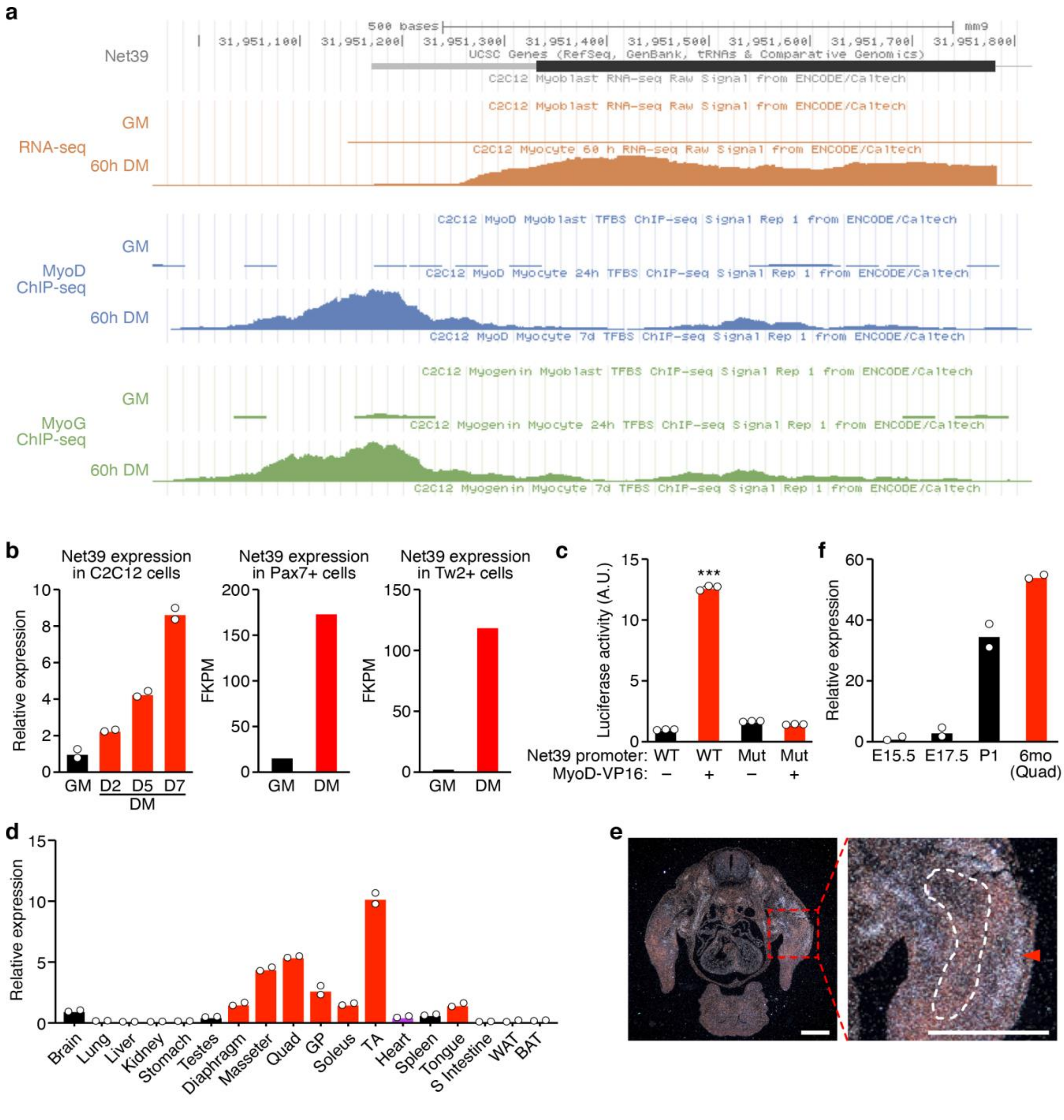

Supplementary Figure 1

Supplementary Figure 1: Muscle expression of Net39.

(**a**) Genome browser track (ENCODE) for *Net39/Plpp7* gene locus with RNA-seq and MyoD and MyoG ChIP-seq from C2C12 muscle cells in growth media (GM) and 60h after induction of differentiation in differentiation media (DM). (**b**) qRT-PCR analysis of Net39 expression during C2C12 differentiation GM and different days in DM. Levels are relative to expression in GM. n=2 technical replicates (left). Net39 expression in satellite cell-derived Pax7$^+$ primary myoblasts before and after 48h of differentiation, as determined by RNA-seq (center). Net39 expression in interstitial myogenic precursor cells (Tw2$^+$) before and after 48h of differentiation, as determined by RNA-seq (right). FPKM: Fragments per kilobase million. (**c**) Luciferase assays with Net39 promoter. A 442bp fragment upstream of Net39 ORF was cloned into a luciferase reporter (WT) or with the E-boxes within mutated (Mut). Luciferase activity was measured in the presence or absence of MyoD-VP16. p=0.0001. n= 3 independent experiments. Data are presented as mean values +/- SEM. Statistical comparisons between groups were evaluated by unpaired and two-sided Student's t-test. (**d**) qRT-PCR analysis of Net39 transcript in adult mouse tissues. Expression is normalized to its expression in brain. Red bars indicate skeletal muscle tissues. Purple bar represents heart tissue. Black bars indicate non-muscle tissues. TA: Tibialis Anterior. WAT: White Adipose Tissue. BAT: Brown Adipose Tissue. n=2 technical replicates. (**e**) In-situ hybridization of Net39 transcript in a transverse section of a mouse at embryonic day 12.5. The area indicated within the white dashed line corresponds to cartilage. Arrowhead denotes positive signal in muscle. Scale bars: 500μm. (**f**) qRT-PCR analysis of Net39 transcript in skeletal muscle during development (black bars) and in adult quadriceps (red bar)**.** Levels are relative to expression at E15.5. n=2 technical replicates. Source data are provided as a Source Data file.

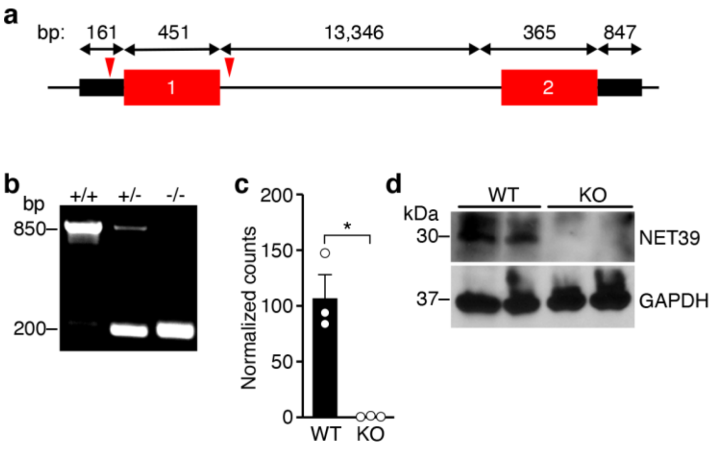

Supplementary Figure 2

Supplementary Figure 2: Knockout strategy for Net39.

(**a**) Gene structure of *Net39/Plpp7* and knock-out strategy. Red arrowheads indicate the locations of the sgRNAs used. Boxes represent exons. Black box denotes untranslated region and red box indicates open reading frame. (**b**) Genotyping of Net39 KO mice by PCR. KO mice have a deletion of 559 base pairs (bp). Three independent experiments with different samples were performed. (**c**) RNA-seq analysis showing loss of Net39 RNA in postnatal day 17 (P17) quadriceps of Net39 KO mice. n= 3 WT and KO mice. *p=0.0322. Data are presented as mean values +/- SEM. (**d**) Western blot analysis showing loss of Net39 protein in P17 quadriceps of Net39 KO mice. GAPDH is a loading control. Two independent experiments were performed. Source data are provided as a Source Data file.

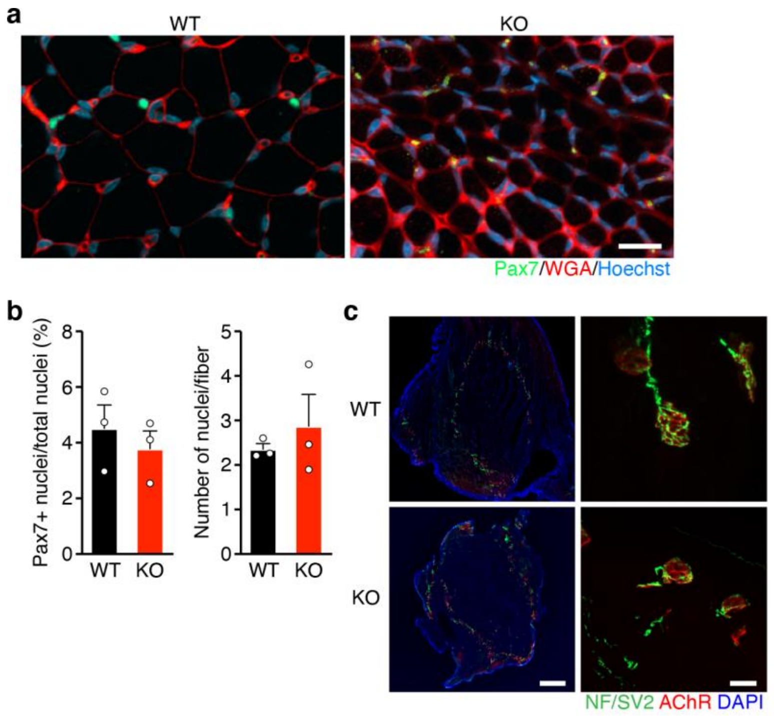

Supplementary Figure 3

Supplementary Figure 3: Analysis of Net39 KO muscles

(**a**) Immunohistochemistry of WT and Net39 KO quadriceps sections at P17 showing the satellite cell marker Pax7 (green). Sections were co-stained with wheat germ agglutinin (WGA) (red), and DAPI (blue). Scale bar: 20μm. (**b**) Quantification of the percentage of $Pax7^{+}$ nuclei relative to the total number of nuclei, and the total number of nuclei/fiber in transverse sections of muscle fibers. n=3 WT and KO mice. Data are presented as mean values +/- SEM. (**c**) Immunofluorescence of neuromuscular junction in transverse quadriceps sections at P17 shows no differences between WT and Net39 KO muscles. Sections were stained for the axons and nerve terminals (NF, SV2) and acetylcholine receptor (AChR) (bungarotoxin). Scale bars: 500μm (left) and 10μm (right). Source data are provided as a Source Data file.

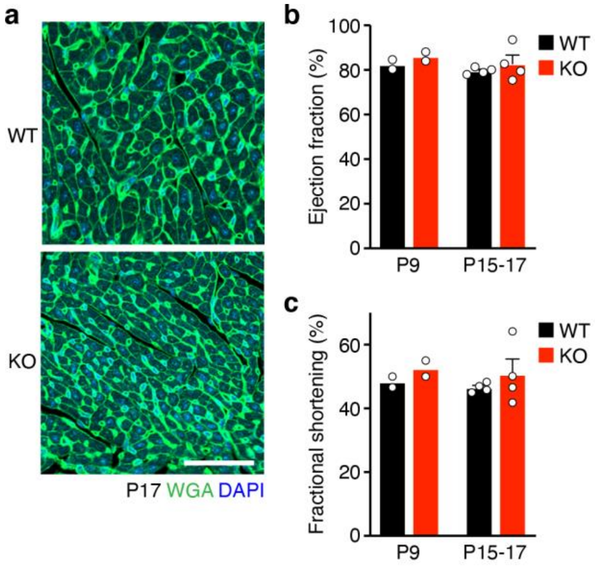

Supplementary Figure 4

Supplementary Figure 4: Histological and functional analyses of Net39 KO hearts. (**a**) Immunohistochemistry of WT and Net39 KO heart sections at P17 showing cardiomyocyte diameters outlined with WGA (green). Scale bar: 50μm. WT and Net39 KO cardiac function was evaluated at P17 by echocardiography and is represented as (**b**) ejection fraction (**c**) and fractional shortening. n=2 for WT and KO mice at P9 and n=4 for WT and KO mice at P17. Data are presented as mean values +/- SEM for n>2. Source data are provided as a Source Data file.

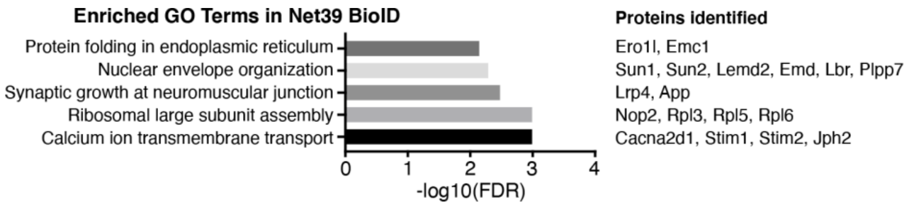

Supplementary Figure 5

Supplementary Figure 5: Net39 interacts with nuclear envelope proteins.

Net39 binding partners were identified by proximity biotinylation (BioID) and enriched Gene ontology terms among the most abundant 50 proteins were determined by STRING analysis. (FDR<0.05).

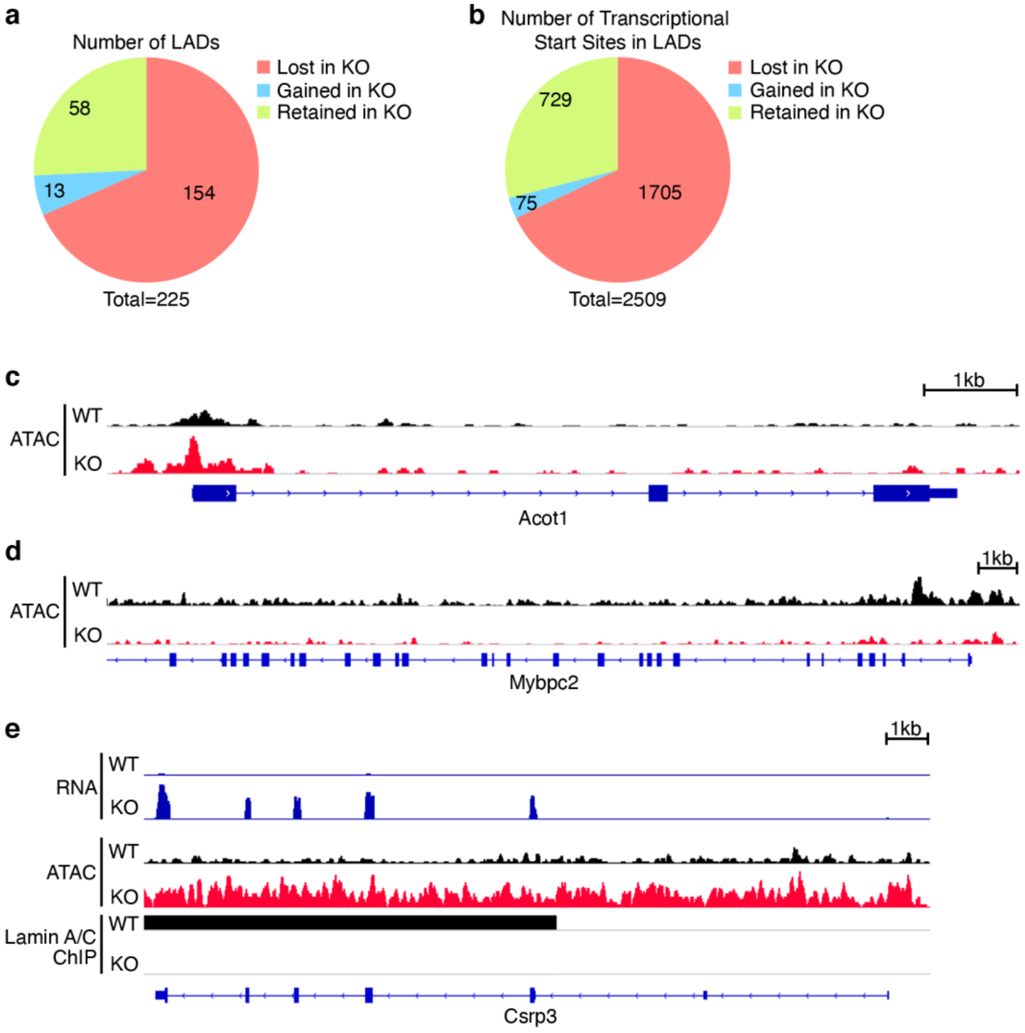

Supplementary Figure 6

Supplementary Figure 6: Analysis of Lamin-Asociated Domains and chromatin accessibility in Net39 KO muscles.

(**a**) Chart illustrating the number of Lamin-Associated Domains (LAD) that are lost (red), gained (blue), or retained (green) from Lamin A/C Chromatin Immunoprecipitation sequencing (ChIP-seq) in Net39 KO quadriceps compared to WT at P17. (**b**) Chart illustrating the number of transcriptional start sites (TSS) within LADs that were lost (red), gained (blue), or retained (green) in Net39 KO quadriceps compared to WT. n=2 WT and KO mice. (**c**) ATAC-seq genome browser shot for Acyl-CoA thioesterase 1 (*Acot1)*, an upregulated gene in Net39 KO muscles. WT: black, KO: red. (**d**) ATAC-seq genome browser shot for Myosin binding protein C2 (*Mybpc2*), a downregulated gene in Net39 KO muscles. WT: black, KO: red. (**e**) Distributions of RNA-seq, ATAC-seq, and Lamin A/C ChIP-seq peaks at the Cysteine and glycine rich protein 3 (*Csrp3)* locus in WT and KO muscles. WT: black, KO: red for ATAC-seq and Lamin A/C ChIP-seq.

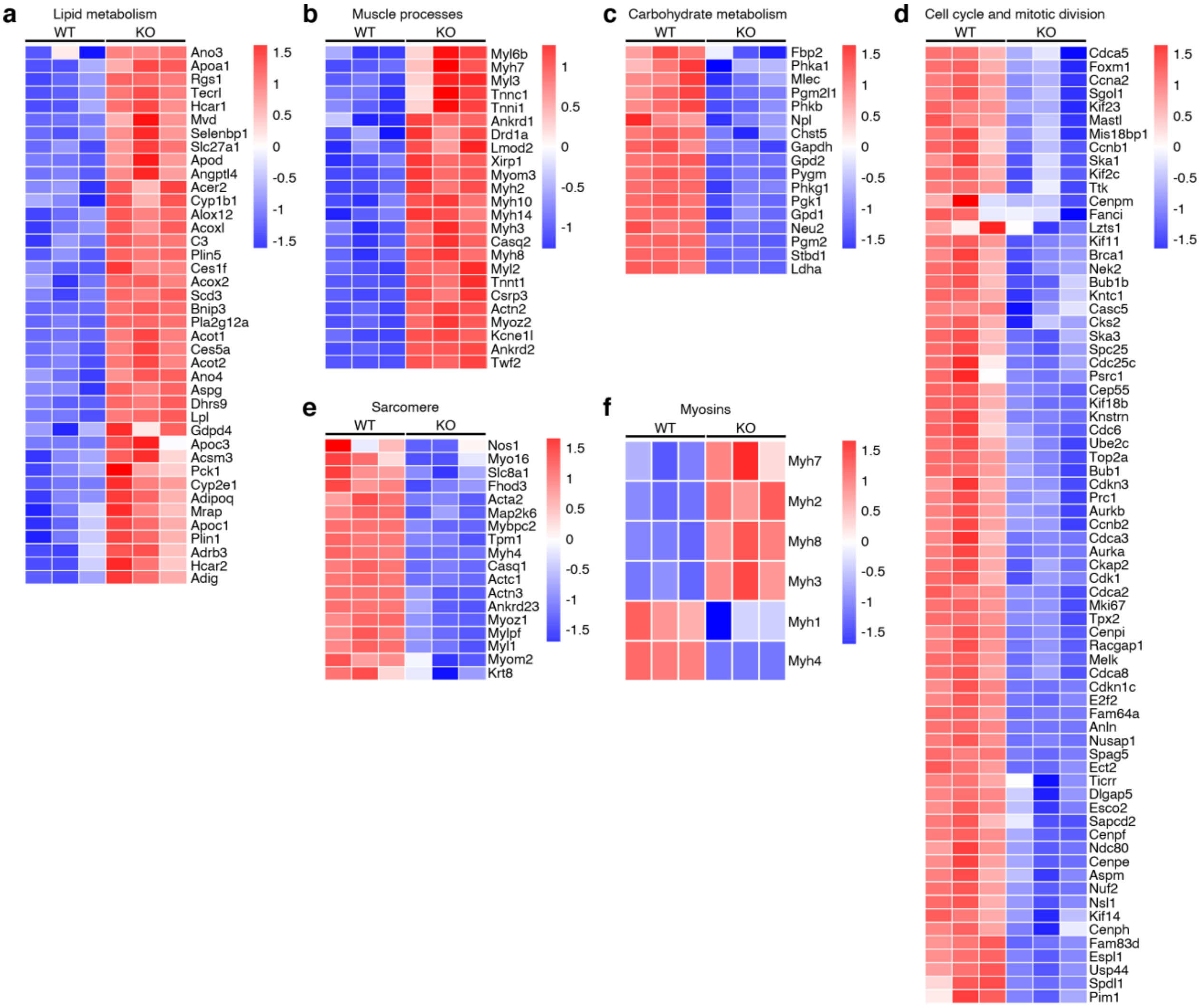

Supplementary Figure 7

Supplementary Figure 7: RNA-seq analysis of Net39 KO muscles at P17.

Heatmaps showing the z-scores for transcript levels of selected genes as determined by RNA-seq and GO pathway analysis. n=3 mice per group. (**a**) Upregulated genes involved in lipid metabolism. (**b**) Upregulated genes involved in muscle processes. (**c**) Downregulated genes involved in carbohydrate metabolism. (**d**) Downregulated genes involved in cell cycle and mitotic division. (**e**) Downregulated genes of the sarcomere. (**f**) Heatmap of myosin genes. Color scale represents Z-score.

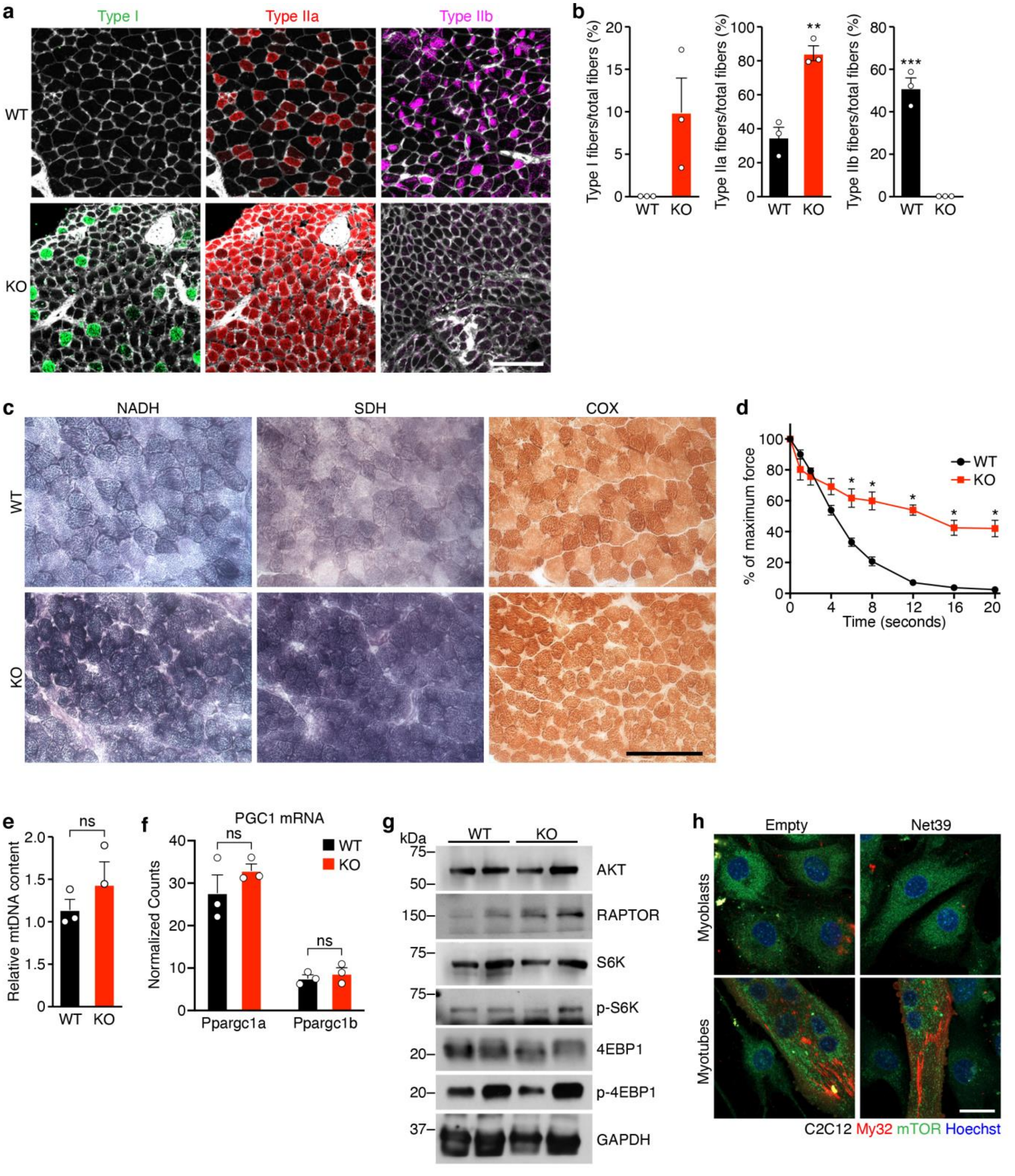

Supplementary Figure 8

Supplementary Figure 8: Net39 KO mice exhibit a shift in oxidative metabolism.

(**a**) Immunohistochemistry of WT and Net39 KO quadriceps sections at P17 showing type I (green), type IIa (red), and type IIb (purple) myofibers. Sections were co-stained with wheat germ agglutinin (WGA) (white). Scale bar: 100μm. (**b**) Quantification of the percentage of type I (p=0.0695) IIa (p=0.025), and IIb (p=0.0004) myofibers relative to the total number of myofibers. n=3 WT and KO mice. Data are presented as mean values +/- SEM. (**c**) Staining of WT and Net39 KO quadriceps at P17 for nicotinamide adenine dinucleotide (NADH), succinic dehydrogenase (SDH) and cytochrome C oxidase (COX). Scale bar: 100μm. (**d**) Ex vivo contraction assay to measure fatigue resistance of EDL muscle. n=8 mice per group. Data are presented as mean values +/- SEM. From left to right, p-values are: 0.24946, 0.56978, 0.05311, 0.01086, 0.00263, 0.00023, 0.00293 and 0.00448. (**e**) Quantification of mtDNA content of WT and Net39 KO quadriceps by qPCR analysis. n= 3 WT and KO mice. Data are presented as mean values +/- SEM. (**f**) RNA-seq analysis showing no changes in PGC1α or PGC1β transcript levels in Net39 KO P17 quadriceps. n= 3 WT and KO mice. Data are presented as mean values +/- SEM. (**g**) Western blot analysis showing protein levels of mTOR signaling components: AKT, RAPTOR, S6K, p-S6K, 4EBP1, p-4EBP1 in P17 quadriceps muscle lysates from WT and Net39 KO mice. GAPDH is a loading control. Two independent experiments were performed. (**h**) Immunofluorescence of mTOR shows no changes upon Net39 overexpression in vitro. Control and Net39 overexpressing C2C12 myotubes and myoblasts were stained for mTOR and My32, a marker of myoblast differentiation. Scale bar: 10μm. Two independent experiments were performed. All statistical comparisons between groups were evaluated by unpaired and two-sided Student's t-test. Source data are provided as a Source Data file.

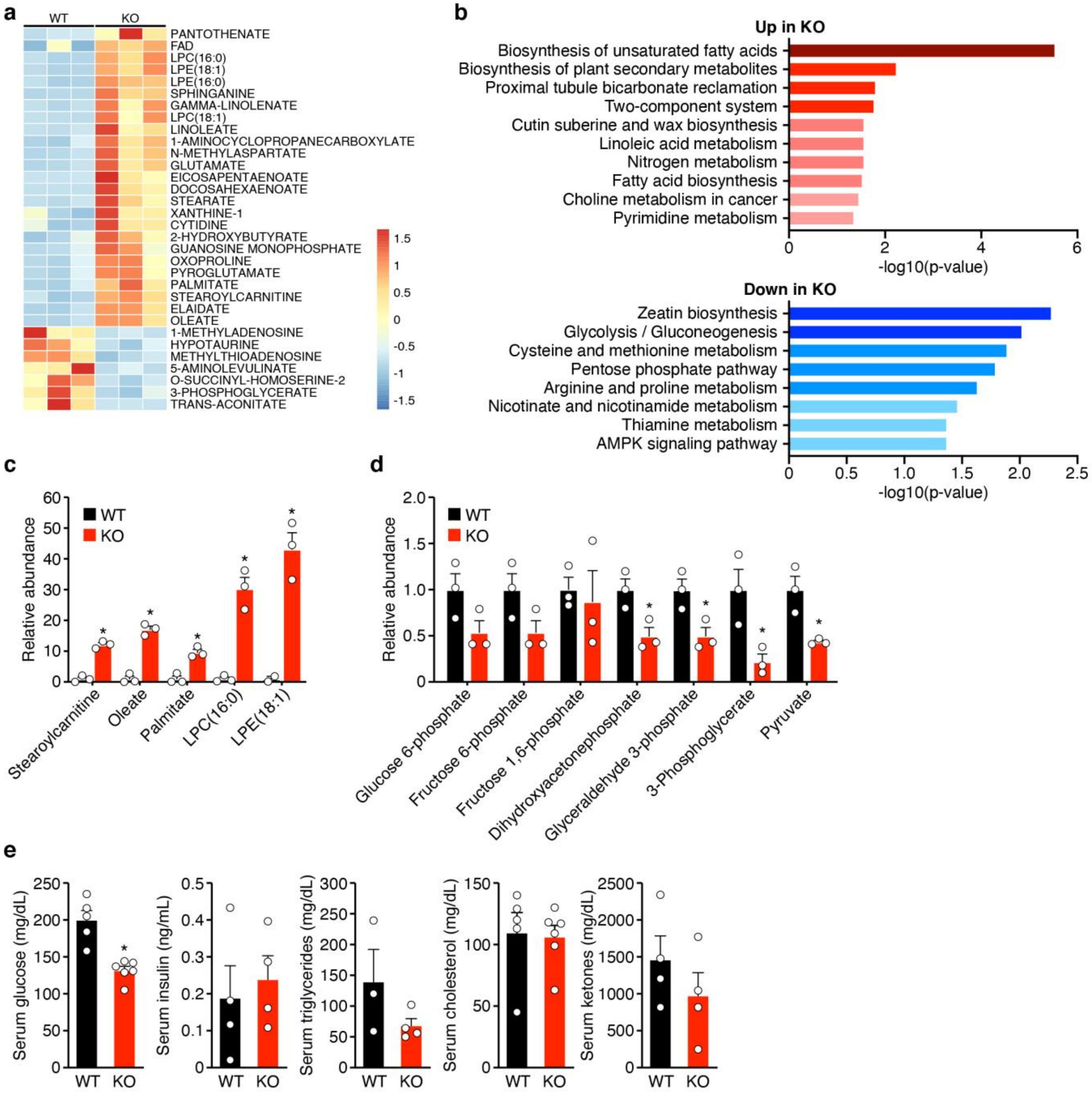

Supplementary Figure 9

Supplementary Figure 9: Metabolic changes in Net39 KO muscles.

(**a**) Heatmap showing upregulated and downregulated metabolites in Net39 KO quadriceps at P17, as analyzed by targeted metabolomics. n=3 mice per group. Color scale represents Z-score. (**b**) GREAT analysis of metabolic pathways enriched among upregulated (top) and downregulated (bottom) metabolites in Net39 KO quadriceps. (**c**) Relative levels of selected elevated fatty acids and lysophospholipids in Net39 KO quadriceps at P17**.** WT was used as reference. From left to right, p-values are: 0.00014, 0.00019, 0.00160, 0.00679 and 0.00704. n= 3 WT and KO mice. Data are presented as mean values +/- SEM. (**d**) Levels of detected glycolysis intermediates in Net39 KO quadriceps at P17. WT was used as reference. From left to right, p-values are: 0.05179, 0.05179, 0.36984, 0.01466, 0.01590, 0.02820 and 0.02859. n= 3 WT and KO mice. Data are presented as mean values +/- SEM. (**e**) Serum levels of the indicated parameters were measured using VITROS clinical diagnostics in WT and Net39 KO mice at P17: glucose (n= 5 WT and 6 KO mice, p=0.0007), insulin (n= 4 WT and 4 KO mice, p=0.6612), triglycerides (n= 3 WT and 3 KO mice, p=0.1843), cholesterol (n= 5 WT and 5 KO mice, p=0.8578), and ketones (n= 4 WT and 4 KO mice, p=0.3206). Data are presented as mean values +/- SEM. All statistical comparisons between groups were evaluated by unpaired Student’s t-test. Source data are provided as a Source Data file.

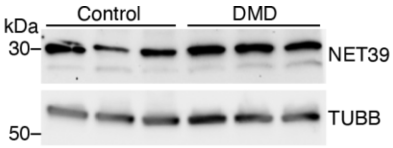

Supplementary Figure 10

Supplementary Figure 10: Net39 protein is not downregulated in Duchenne muscular dystrophy (DMD) mice.

Western blot analysis showing Net39 protein expression of soleus muscle lysates from age-matched WT control and *Dmd* exon 44 deletion (DMD) mice. Tubulin was used as a loading control. n=3 mice each. Source data are provided as a Source Data file.
