# Supplementary material for: The nuclear envelope protein Net39 is essential for muscle nuclear integrity and chromatin organization
Source: Nat Commun. 2021 Jan 29;12:690. doi: 10.1038/s41467-021-20987-x (PMC7846557; doi:10.1038/s41467-021-20987-x)
Supplement: Supplementary file 2 — Description of Additional Supplementary Files [file 41467_2021_20987_MOESM2_ESM.pdf]

Description of Additional Supplementary Files

File Name: Supplementary Data 1

Description: **Net39 BioID in C2C12 myotubes.**

Two independent runs of Net39 BioID were performed in C2C12 myotubes overexpressing Net39-miniTurbo. Samples were incubated in 500uM Biotin for 4h (Biotin) or in regular media (Ctrl) before streptavidin pulldown was performed. Table provides PSM (peptide-spectrum match), abundance, and enrichment for each protein. Proteins without quantifiable abundance were excluded.

File Name: Supplementary Data 2

Description: **Net39 muscle metabolomics**

Metabolomics analysis was performed on WT and Net39 KO quadriceps at P17. The values indicated in the table correspond to peak intensity for each metabolite in the different samples.

File Name: Supplementary Data 3

Description: **Primer list**

All the primers used in the study are included in the table.
