# Supplementary material for: The nuclear envelope protein Net39 is essential for muscle nuclear integrity and chromatin organization
Source: Nat Commun. 2021 Jan 29;12:690. doi: 10.1038/s41467-021-20987-x (PMC7846557; doi:10.1038/s41467-021-20987-x)
Supplement: Supplementary file 6 — Reporting Summary [file 41467_2021_20987_MOESM6_ESM.pdf]

nature research

Corresponding author(s): Eric N. Olson and Ning Liu

Last updated by author(s): Dec 2, 2020

Reporting Summary

Nature Research wishes to improve the reproducibility of the work that we publish. This form provides structure for consistency and transparency in reporting. For further information on Nature Research policies, see our Editorial Policies and the Editorial Policy Checklist.

Statistics

For all statistical analyses, confirm that the following items are present in the figure legend, table legend, main text, or Methods section.

n/a	Confirmed	
☐	☒	The exact sample size (n) for each experimental group/condition, given as a discrete number and unit of measurement
☐	☒	A statement on whether measurements were taken from distinct samples or whether the same sample was measured repeatedly
☐	☒	The statistical test(s) used AND whether they are one- or two-sided<br>*Only common tests should be described solely by name; describe more complex techniques in the Methods section.*
☒	☐	A description of all covariates tested
☐	☒	A description of any assumptions or corrections, such as tests of normality and adjustment for multiple comparisons
☐	☒	A full description of the statistical parameters including central tendency (e.g. means) or other basic estimates (e.g. regression coefficient) AND variation (e.g. standard deviation) or associated estimates of uncertainty (e.g. confidence intervals)
☐	☒	For null hypothesis testing, the test statistic (e.g. F, t, r) with confidence intervals, effect sizes, degrees of freedom and P value noted<br>*Give P values as exact values whenever suitable.*
☒	☐	For Bayesian analysis, information on the choice of priors and Markov chain Monte Carlo settings
☒	☐	For hierarchical and complex designs, identification of the appropriate level for tests and full reporting of outcomes
☒	☐	Estimates of effect sizes (e.g. Cohen's d, Pearson's r), indicating how they were calculated

Our web collection on statistics for biologists contains articles on many of the points above.

Software and code

Policy information about availability of computer code

Data collection	MultiQuant software v.2.1 (Applied Biosystems SCIEX), Proteome Discoverer v2.2 (Thermofisher)
Data analysis	Picard tools (v.2.10.3). Enriched Domain Detector (v.1.0), Bedtools (v.2.29.0). Bowtie2 (version 2.3.4.3), R package DEseq version 3.8, Limma R Bioconductor package, HISAT2 aligner (v2.1.0).

For manuscripts utilizing custom algorithms or software that are central to the research but not yet described in published literature, software must be made available to editors and reviewers. We strongly encourage code deposition in a community repository (e.g. GitHub). See the Nature Research guidelines for submitting code & software for further information.

Data

Policy information about availability of data

All manuscripts must include a data availability statement. This statement should provide the following information, where applicable:

- Accession codes, unique identifiers, or web links for publicly available datasets
- A list of figures that have associated raw data
- A description of any restrictions on data availability

Proteomics and metabolomics data provided in Supplementary Data 1 and 2. RNA-seq, ATAC-seq and ChIP-seq datasets have been uploaded to GEO (accession: GSE154850) and are publicly available at [https://www.ncbi.nlm.nih.gov/geo/query/acc.cgi?acc=GSE154850].
No restrictions on data availability.
Figures with associated Raw data: Fig.1-4, Supplementary Figures 1-9

Field-specific reporting

Please select the one below that is the best fit for your research. If you are not sure, read the appropriate sections before making your selection.

☒ Life sciences ☐ Behavioural & social sciences ☐ Ecological, evolutionary & environmental sciences

For a reference copy of the document with all sections, see nature.com/documents/nr-reporting-summary-flat.pdf

Life sciences study design

All studies must disclose on these points even when the disclosure is negative.

Sample size	A sample size of N=3 for WT vs KO comparisons was used throughout the study. No sample size calculation was performed beforehand.
Data exclusions	For RNA-seq and ATAC-seq, no data was excluded. For Lamin A ATAC-seq, KO1 sample was excluded due to poor quality.
Replication	Experiments were repeated multiple times and results were replicated by two independent investigators. Information for each experiment replication is included in the manuscript.
Randomization	Randomization was not considered in the study.
Blinding	Investigators were not blinded when performing sample preparation or analysis.

Reporting for specific materials, systems and methods

We require information from authors about some types of materials, experimental systems and methods used in many studies. Here, indicate whether each material, system or method listed is relevant to your study. If you are not sure if a list item applies to your research, read the appropriate section before selecting a response.

Materials & experimental systems

n/a	Involved in the study	
☐	☒	Antibodies
☐	☒	Eukaryotic cell lines
☒	☐	Palaeontology and archaeology
☐	☒	Animals and other organisms
☐	☒	Human research participants
☒	☐	Clinical data
☒	☐	Dual use research of concern

Methods

n/a	Involved in the study	
☐	☒	ChIP-seq
☒	☐	Flow cytometry
☒	☐	MRI-based neuroimaging

Antibodies

Antibodies used	Primary and conjugated Alexa Fluor secondary antibodies (ThermoFisher) were used at 1:200 dilution. The following antibodies and conjugated fluorophores were used: Net39 (Sigma, HPA070252), Sun2 (Sigma, MABT880), Lmna (Abcam, ab26300), Lamin B1 (Abcam, ab16048) Lamin A/C (Santa Cruz, sc-7292 X), Lemd2 (Sigma, HPA017340), Emerin (Santa Cruz, sc-25284), Myh7 (Santa Cruz, sc-53089), Myh2 (Santa Cruz, sc-53096), Myh4 (Proteintech, 20140-1-AP), Pax7 (DSHB, PAX7-c), AKT (Cell Signaling, 9272), RAPTOR (Cell Signaling, 2280), S6K (Cell Signaling, 9202), p-S6K (Thr389) (Cell Signaling, 9234), 4EBP1 (Cell Signaling, 9452) and p-4EBP1 (Ser65) (Cell Signaling, 9451), mTOR (Cell Signaling, 2983), My32 (Sigma, M4276), GAPDH (Sigma, MAB374), Tubulin (Abcam, ab6046), Histone H3 (Cell Signaling, 9715S) Phalloidin-488 (ThermoFisher, A12379), Wheat-germ agglutinin-488 (ThermoFisher, W11261), HRP-conjugated streptavidin (Thermofisher, N100), SV2 (DSHB, SV2-c), Neurofilament (DSHB, 2H3-c), α-Bungarotoxin-555 (ThermoFisher, B35451).
Validation	Net39 Antibody was validated using Net39 KO samples (Supplementary Fig. 2d). All other antibodies were previously validated by the provider and used extensively in prior publications.

Eukaryotic cell lines

Policy information about cell lines

Cell line source(s)	C2C12 (ATCC, CRL-1772) Platinum E cells (Cell Biolabs, NC0066908) , HEK 293T (ATCC CRL-3216)
Authentication	None of the cell lines were authenticated.
Mycoplasma contamination	Cells were negative for mycoplasma contamination.

Commonly misidentified lines (See ICLAC register)	None.

Animals and other organisms

Policy information about studies involving animals; ARRIVE guidelines recommended for reporting animal research

Laboratory animals	B6C3F1 mice (5 weeks old) were used to generate Net39 KO mouse line and subsequently crossed to C57BL6N mice. Samples were collected at 9 or 17 days of age.
Wild animals	The study did not involve samples collected from wild animals.
Field-collected samples	The study did not involve samples collected from the field.
Ethics oversight	All animal procedures were approved by the Institutional Animal Care and Use Committee at the University of Texas Southwestern Medical Center.

Note that full information on the approval of the study protocol must also be provided in the manuscript.

Human research participants

Policy information about studies involving human research participants

Population characteristics	The pathology database at UTSW Medical Center was retrospectively reviewed. The database contains 10,070 muscle biopsies from different ages and genders .
Recruitment	All patients with muscle biopsy and genetically confirmed LMNA mutations were included. Normal muscle biopsies from individuals with matched age, gender and biopsy site were included as controls.
Ethics oversight	The use of medical record and human tissues for research purposes was approved by the UTSW Human Subjects Office IRB (protocol no. 012016-082).

Note that full information on the approval of the study protocol must also be provided in the manuscript.

ChIP-seq

Data deposition

☒ Confirm that both raw and final processed data have been deposited in a public database such as GEO.

☒ Confirm that you have deposited or provided access to graph files (e.g. BED files) for the called peaks.

Data access links *May remain private before publication.*	RNA-seq, ATAC-seq and ChIP-seq datasets have been uploaded to GEO (GSE154850) and are publicly available at [https://www.ncbi.nlm.nih.gov/geo/query/acc.cgi?acc=GSE154850]
Files in database submission	RNA-seq, ATAC-seq and ChIP-seq datasets in "GSE154850_RAW.tar"
Genome browser session (e.g. UCSC)	*Provide a link to an anonymized genome browser session for "Initial submission" and "Revised version" documents only, to enable peer review. Write "no longer applicable" for "Final submission" documents.*

Methodology

Replicates	3 biological replicates were used per group (WT or Net39 KO).
Sequencing depth	All reads were single-end sequenced. For the WT1 sample, 44749081 reads were obtained from the Lamin ChIP, 32290069 of which were uniquely mapped, for the input there were 46309062 reads total and 36764106 reads were unique. For WT2 sample, 39002307 reads were obtained from the Lamin ChIP and 15351494 reads were unique. For the WT2 input, 43250224 reads were obtained and 34715496 were unique. For the WT3 sample, 41637542 reads were obtained from the Lamin ChIP and 31967992 were unique. For the WT3 input, 45341782 reads were obtained and 36316225 were unique. For the KO1 sample, 22893973 reads were obtained from the Lamin ChIP and 2685727 were unique. For the KO1 input, 45090387 reads were obtained and 36168413 were unique. For the KO2 sample, 40816758 reads were obtained in total from the Lamin ChIP and 23734555 were unique. For the KO2 input, 44090709 reads were obtained in total and 35490683 reads were unique.
Antibodies	Lamin A/C (Santa Cruz, sc-7292 X)
Peak calling parameters	To detect LAD domains, Enriched Domain Detector (v.1.0) was used with a 10-kb bin size, gap penalty of 10 and FDRadjusted significance threshold of 0.05. Gain, loss and overlapping LADs between WT and KO samples were tallied using bedtools (v.2.29.0).
Data quality	A total of 225 peaks corresonding lamin associated domains (LADs) were detected to be statistically significant enriched over inputs (FDR <0.05).

Software	Picard tools (v.2.10.3). Enriched Domain Detector (v.1.0)Bedtools (v.2.29.0).
